# Supplementary material for: Low Frequency Oscillations in the Medial Orbitofrontal Cortex Mediate Widespread Hyperalgesia Across Pain Conditions
Source: medRxiv. 2025 Jun 16:2025.06.15.25329637. Preprint. [Version 1] doi: 10.1101/2025.06.15.25329637 (PMC12204252; doi:10.1101/2025.06.15.25329637)
Supplement: Supplementary file 1 [file NIHPP2025.06.15.25329637v1-supplement-1.pdf]

medRxiv preprint doi: https://doi.org/10.1101/2025.06.15.25329637; this version posted June 16, 2025. The copyright holder for this preprint (which was not certified by peer review) is the author/funder, who has granted medRxiv a license to display the preprint in perpetuity.
It is made available under a CC-BY 4.0 International license .

Supporting Information for

Low Frequency Oscillations in the Medial Orbitofrontal Cortex Mediate Widespread Hyperalgesia Across Pain Conditions

This file includes: Figures S1 to S3

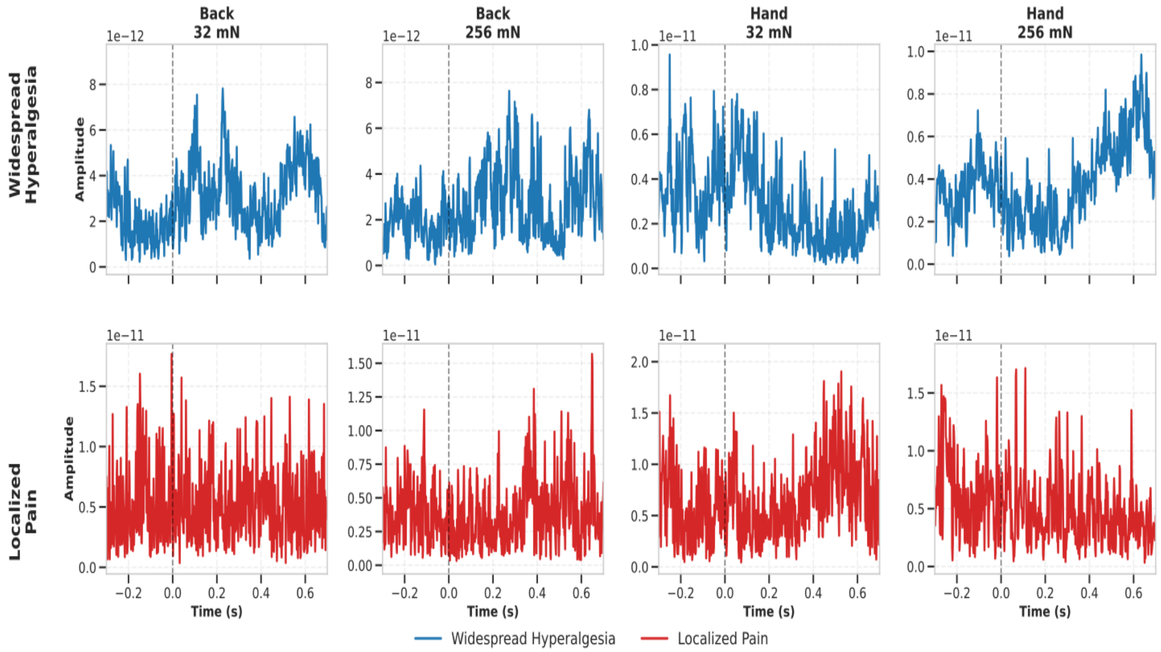

Figure S1. Source-localized EEG traces from the left mOFC in representative cLBP participants. Top row: Source-localized EEG traces from the mOFC (left hemisphere) for a representative participant with widespread hyperalgesia, shown across four stimulus conditions (Back 32 mN, Back 256 mN, Hand 32 mN, Hand 256 mN). **Bottom row**: Traces from a representative participant with localized pain, under the same four stimulus conditions. Epochs were extracted from -0.3 to 0.7 seconds relative to each pinprick stimulus onset. Time-frequency representations (TFRs) of power (1 to 45 Hz) were computed using real and imaginary signal components of the decomposition and transformed into decibel scale. Baseline correction was performed by subtracting the average pre-stimulus power (-0.3 to 0 s) for each trial per frequency. Corrected dB power responses were then averaged across trials per condition to yield subject- and stimulus condition-specific TFRs for each of the four regions of interest (bilateral mOFC and dlPFC).

medRxiv preprint doi: https://doi.org/10.1101/2025.06.15.25329637; this version posted June 16, 2025. The copyright holder for this preprint
(which was not certified by peer review) is the author/funder, who has granted medRxiv a license to display the preprint in perpetuity.
It is made available under a CC-BY 4.0 International license .

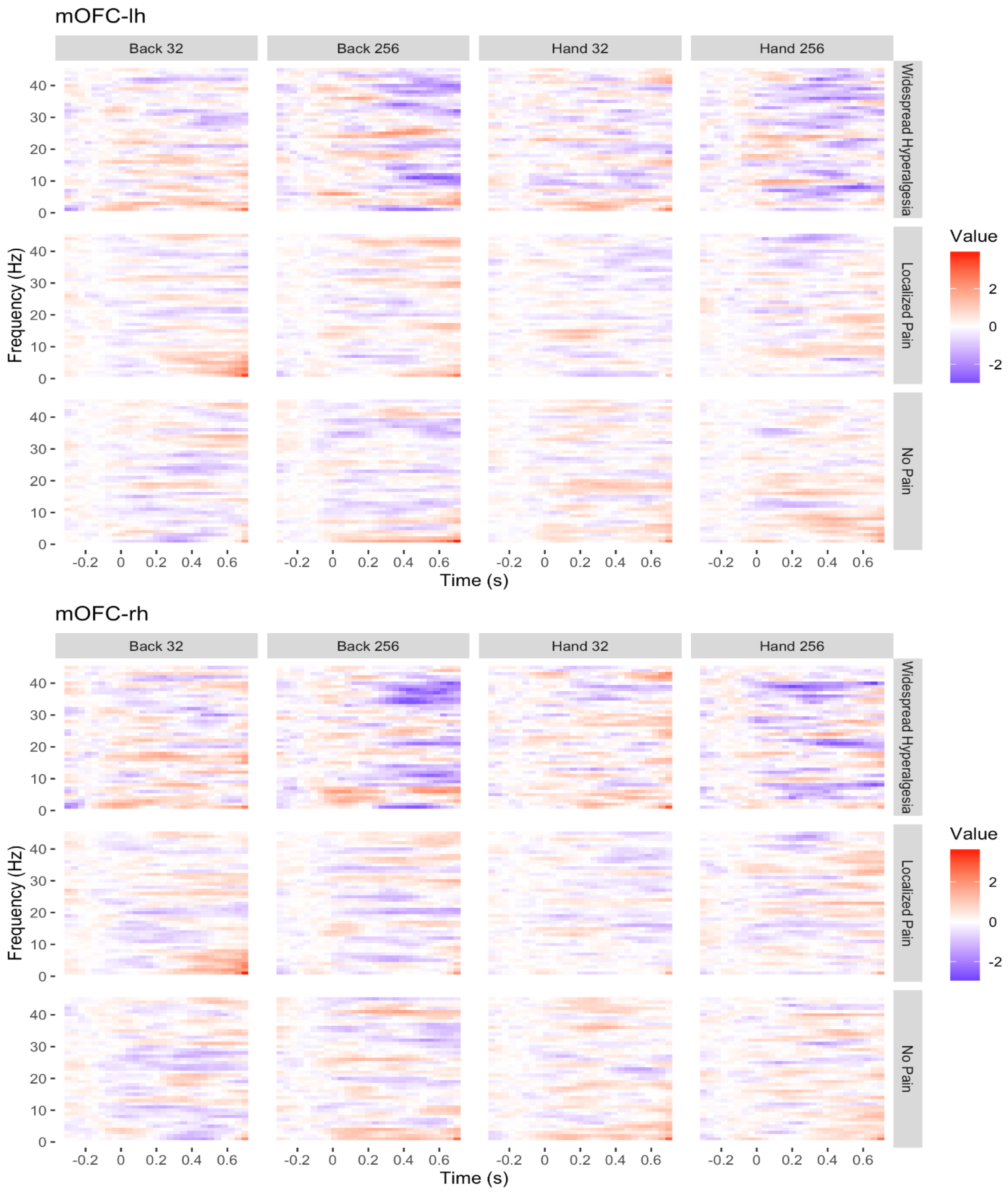

Figure S2. Time-frequency EEG responses in the mOFC by stimulus condition and pain phenotype in the chronic low back pain (cLBP) cohort (n = 67). Group- and stimulus-specific averaged time-frequency representations (TFRs) for the cLBP cohort, averaged across participants within each pain phenotype group (Widespread Hyperalgesia, Localized Pain, No Pain). Top panels show responses from the left mOFC; bottom panels from the right mOFC. Each panel displays baseline-corrected decibel power from -0.3 to 0.7 seconds post-stimulus across four conditions: Back 32 mN, Back 512 mN, Hand 32 mN, and Hand 512 mN.

medRxiv preprint doi: https://doi.org/10.1101/2025.06.15.25329637; this version posted June 16, 2025. The copyright holder for this preprint (which was not certified by peer review) is the author/funder, who has granted medRxiv a license to display the preprint in perpetuity.
It is made available under a CC-BY 4.0 International license .

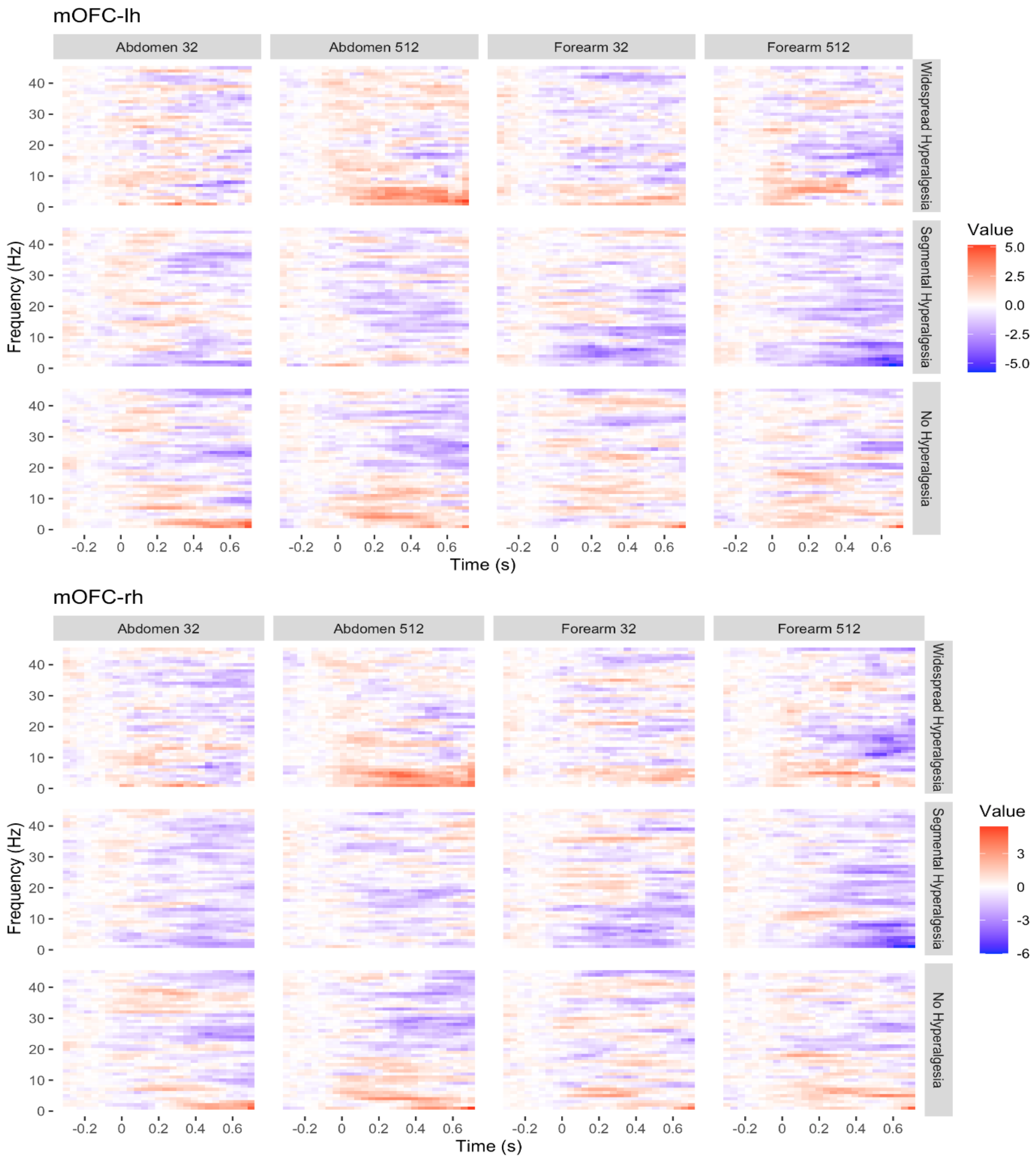

Figure S3. Time-frequency EEG oscillatory responses in the mOFC by stimulus condition and pain phenotype in the chronic pancreatitis (CP) cohort (n = 18). Group-averaged time-frequency representations (TFRs) are shown for each pain phenotype group (Widespread, Segmental, No Hyperalgesia), stratified by stimulation site and intensity: Abdomen 32 mN, Abdomen 512 mN, Forearm 32 mN, and Forearm 512 mN. Each panel displays baseline-corrected power (in decibels) from –0.3 to 0.7 seconds post-stimulus. Top panels reflect responses from the left mOFC; bottom panels from the right mOFC. The widespread hyperalgesia group (top row) exhibited elevated delta/theta (1–7 Hz) power under high-intensity stimulation at the affected site (Abdomen 512 mN), compared to the segmental hyperalgesia group (middle row). The segmental hyperalgesia group showed relatively reduced delta/theta activity at the unaffected site (Forearm 512 mN), suggesting differential neural processing of noxious input across phenotypes.
